# The carbon brace

**DOI:** 10.1186/1748-7161-8-3

**Published:** 2013-02-14

**Authors:** Jean-Claude Bernard, Cyril Lecante, Julie Deceuninck, Gregory Notin, Lydie Journoud, Frederic Barral

**Affiliations:** 1 Orthopaedic Department, Massues Hospital, CMCR des Massues, Lyon, France; 2 Medical doctor, Massues Hospital, CMCR des Massues, Lyon, France; 3 Technician Ortho Prothesist, Lyon, France; 4 Physiotherapist, CMCR des Massues Croix Rouge française, Lyon, France

## Abstract

**Background:**

The CMCR brace (**C**orset **M**onocoque**C**arbone respectant la **R**espiration –which means Monoshell Carbon Brace respecting Breathing) is an innovative brace, used in orthopaedic treatment for progressive thoracic, thoraco-lumbar or combined scoliosis, whatever their etiology. It can be used at the very young age without disrupting the chest growth, but should be kept for reducible scoliosis in older teenagers.

**Brace description and principles:**

The CMCR brace is monoshell while retaining the corrective principle of the polyvalve Lyon brace with one or two supports (brace “pads”) located on hump(s).In contrast to Lyon brace made of plexidur and structured by metal reinforcement with adjustable but fixed localized supports, the CMCR brace is made of polyethylene and carbon with adjustable and mobile supports. This mobility provides a permanent pressure, which varies depending on ribs and spine movements.

The correction is obtained without spinal extension so that each respiratory movement takes part in a gradual return to dorsal kyphosis.

**Results:**

Results were presented in two published analysis:

• In the first retrospective study about 115 patients, French-published in the *Annals of Physical Medicine and Rehabilitation* (2005), the CMCR brace stabilized moderate scoliosis, decreased the vital capacity (VC) of 13% compared to the VC without brace, and did not have sufficient impact on the hump reduction. Treatment had better results when started at Risser 3 or 4 than Risser 0, 1, 2. The brace was then modified to increase the dorsal pad pressure and the location of correction forces was defined more precisely through the use of 3D analysis.

• The second study published in *Scoliosis* (2011) mainly focused on the impact on VC at brace setting up and followed a cohort of 90 patients treated with CMCR. Girls as well as boys increased VC during treatment, and at brace definitive removal, VC had increased of 21% from the initial value, whereas the theoretical VC at the same time rose by 18%.

The difference between the time where the child actually wears its brace and the time asked by the clinician for the brace to be worn is only 1 hour, which means that this brace is accepted by teenagers.

**Conclusions:**

Orthopaedic treatment is still a heavy treatment for teenagers in growth period. This orthosis is designed to partly maintain spine and chest mobility. We hope so to have part in improving life conditions of these teenagers, compared to those treated with rigid braces.

## Introduction

Idiopathic scoliosis affects 2 to 3% of the population, including 0.3 to 0.5% with a scoliosis over than 20°. The Lyon treatment is applied since the 1950s for the conservative treatment in scoliosis over 30° and comes in 2 phases:

**Figure 1:**
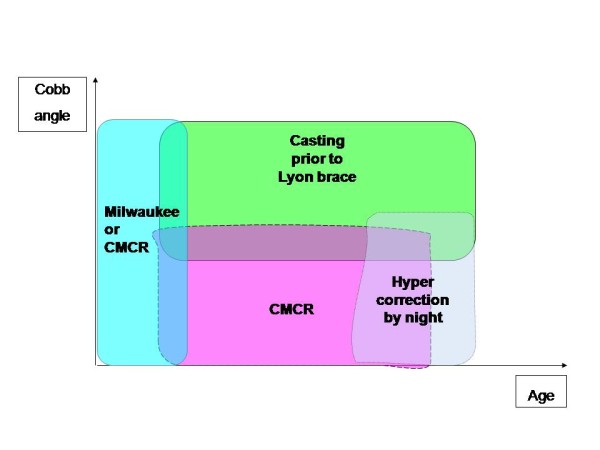
Diagram for brace indications regarding age and Cobb angle.

**Figure 2:**
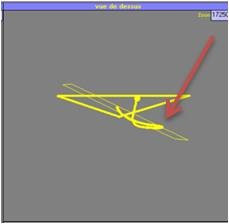
Localization of pressure.

**Figure 3:**
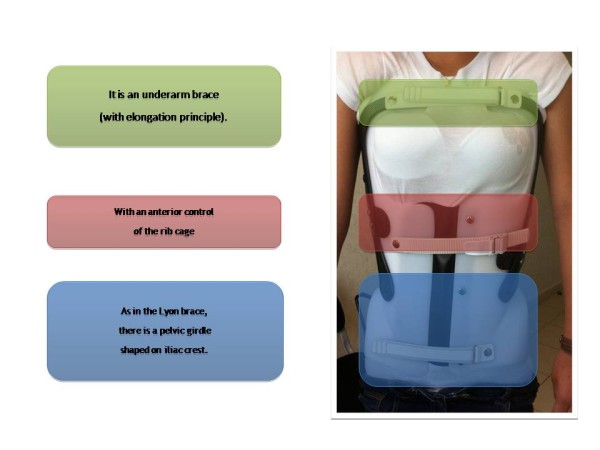
Three kydex support pieces; 2 side ones, and 1 posterior.

**Figure 4:**
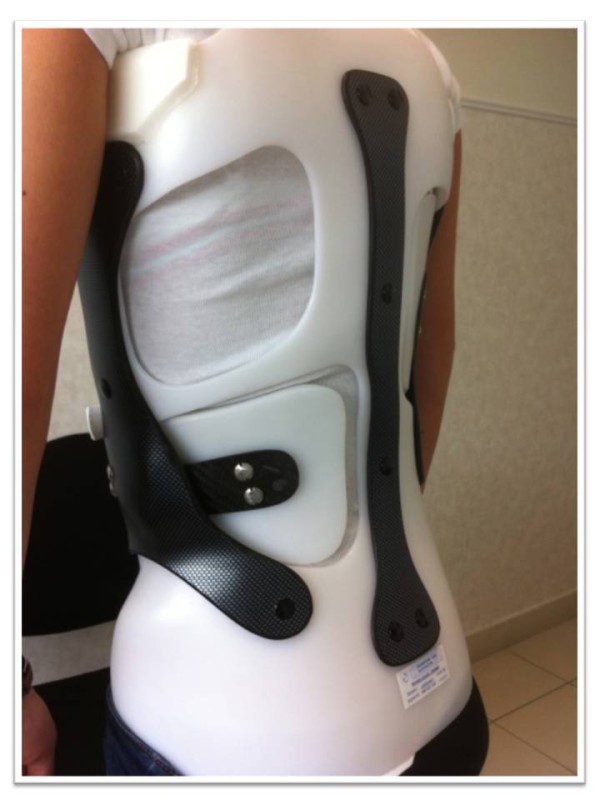
Front view of the main shell.

**Figure 5:**
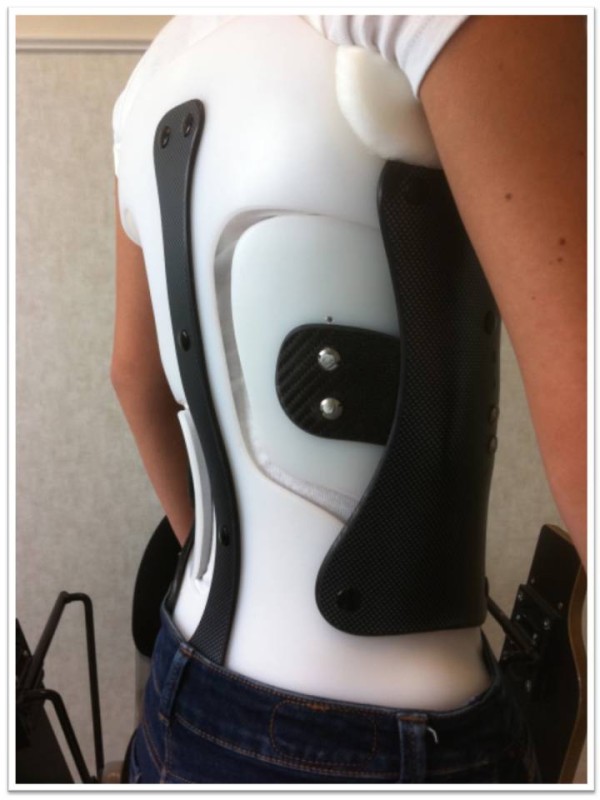
Left lumbar pad.

**Figure 6:**
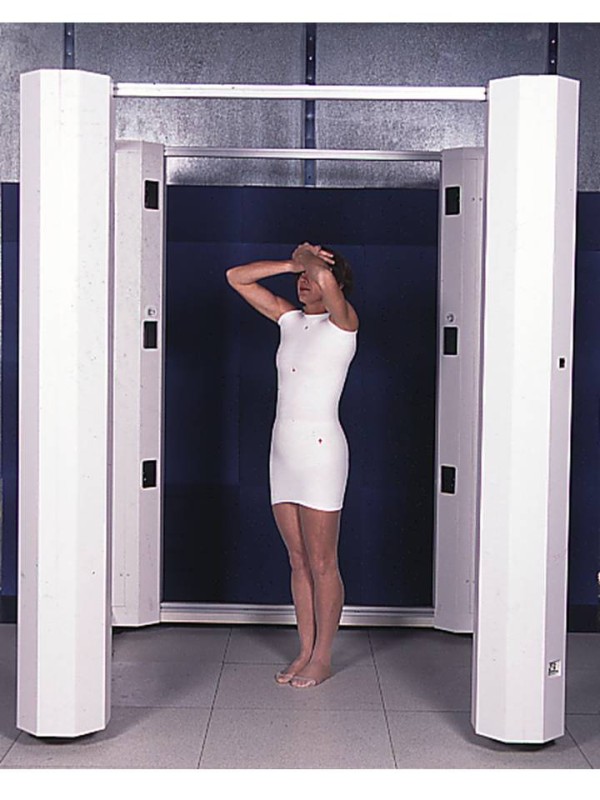
Right thoracic pad.

**Figure 7:**
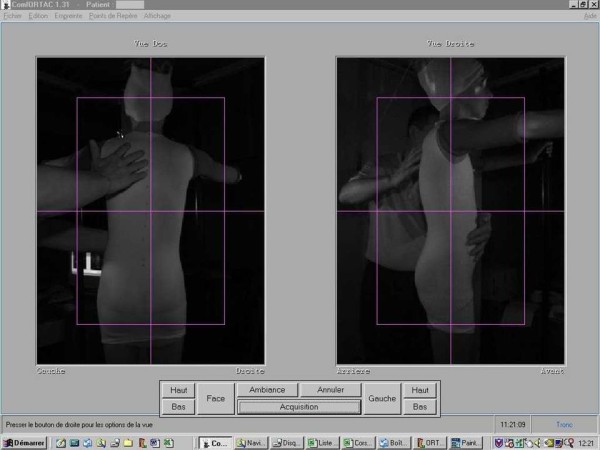
Three-dimensional scanning unit (ORTEN optical sensor).

**Figure 8:**
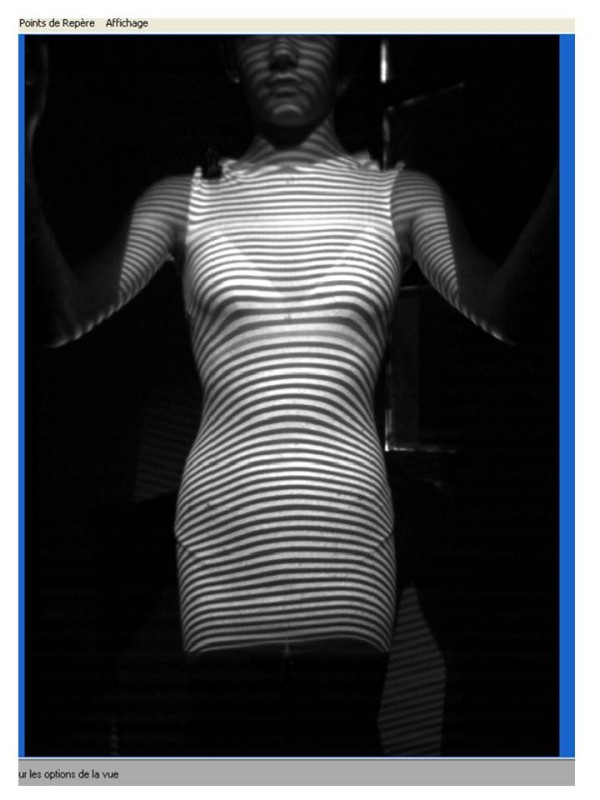
Patient positioning during optical print acquisition.

**Figure 9:**
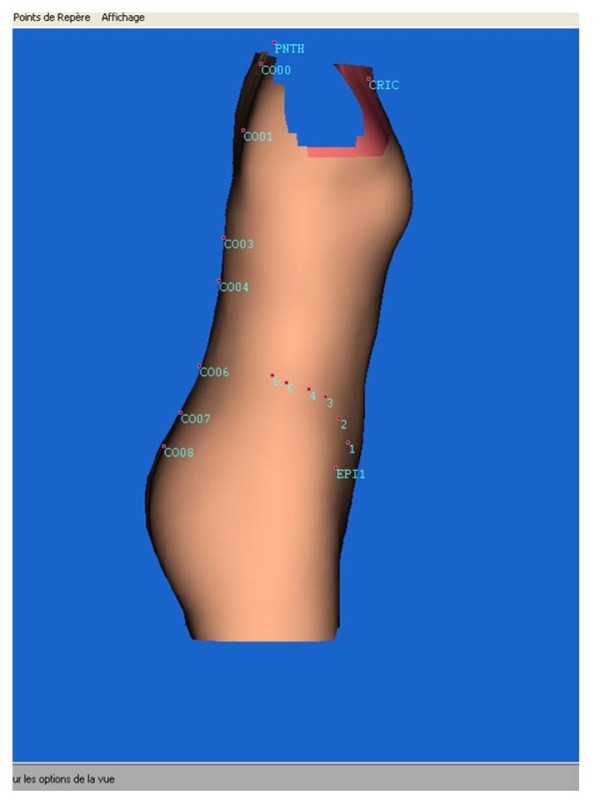
Fringe used for the 3D thorax reconstruction.

**Figure 10:**
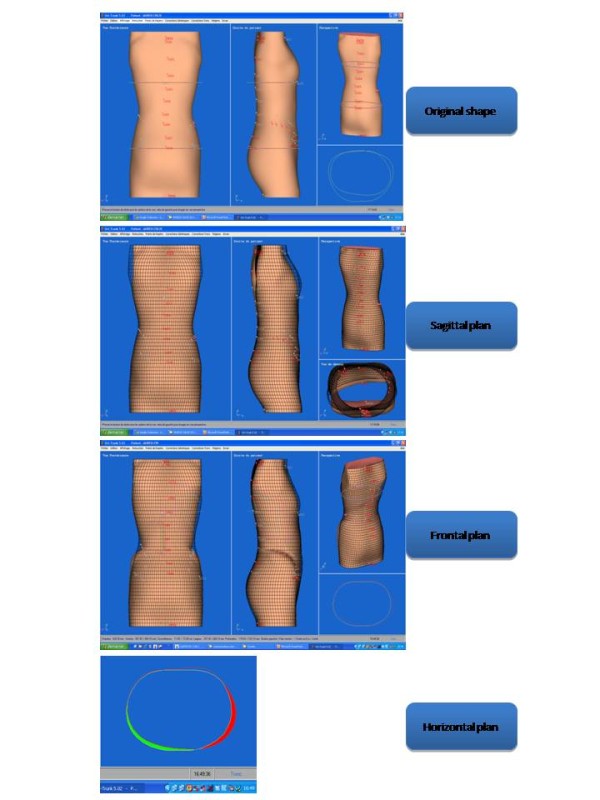
3D thorax reconstruction.

**Figure 11:**
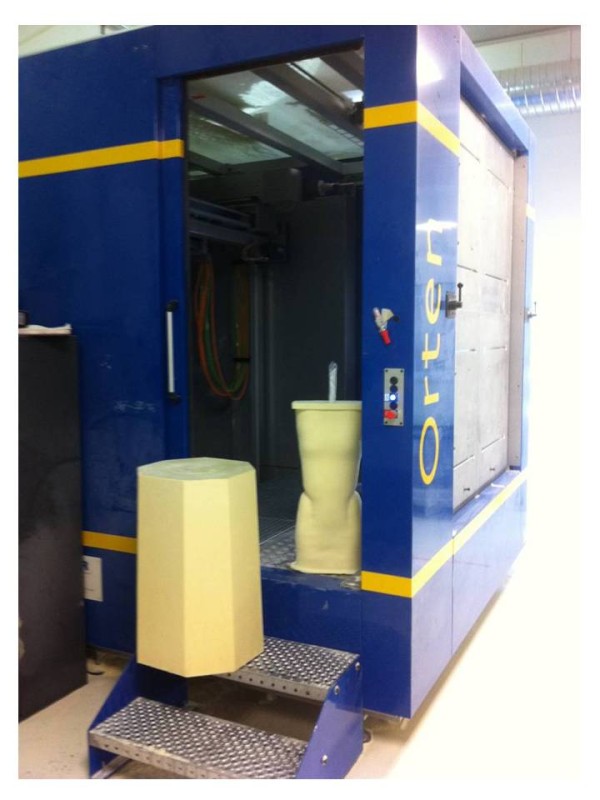
Milling different steps in rectifying positive.

**Figure 12:**
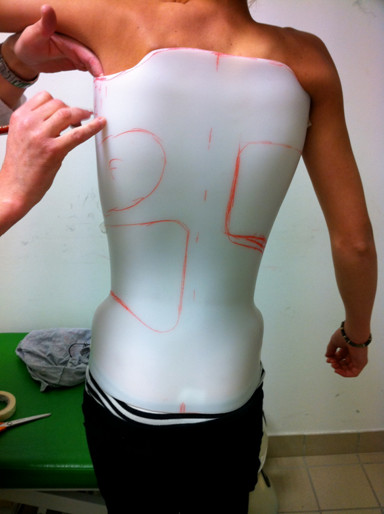
Numerically controlled milling machine.

**Figure 13:**
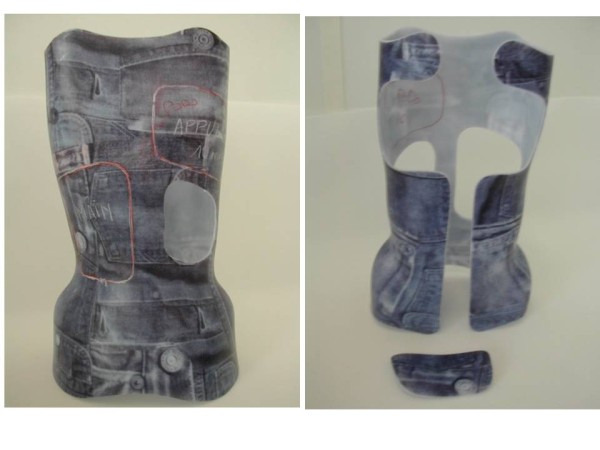
Fitting thepolyethylene shell.

**Figure 14:**
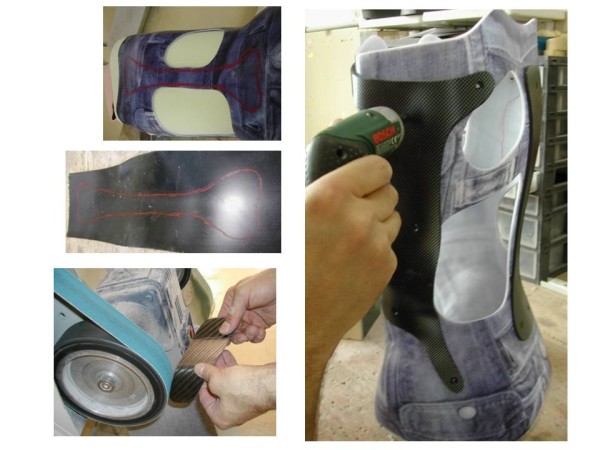
Posterior and front views of the shell after fitting.

**Figure 15:**
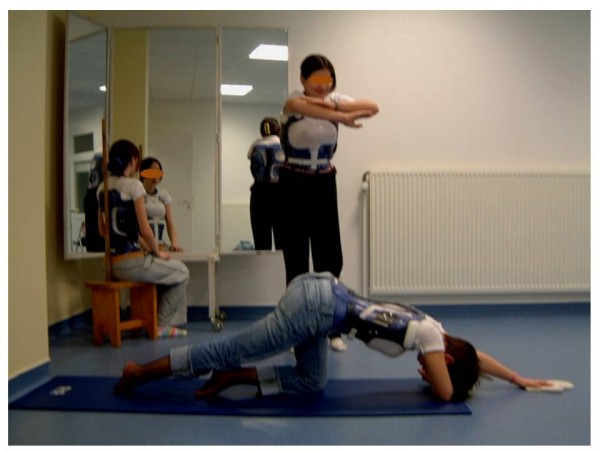
Different steps in CMCR assembly.

**Figure 16:**
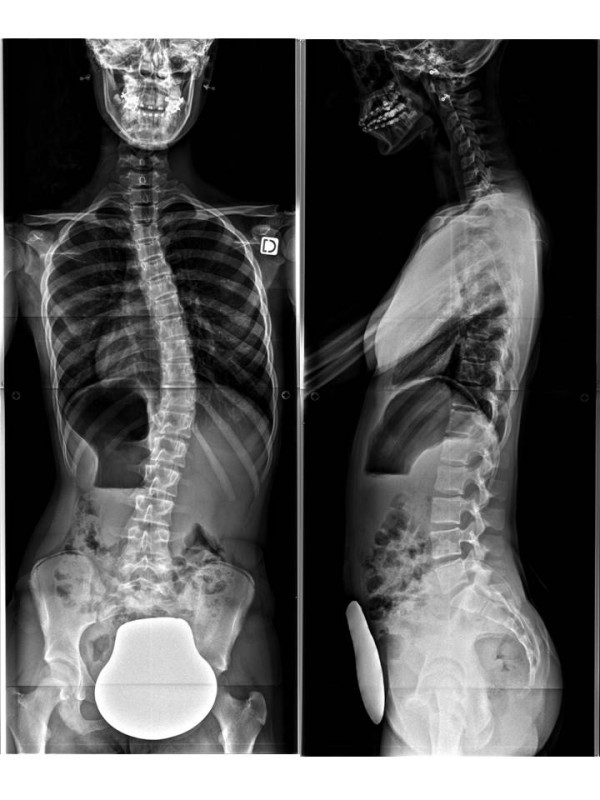
Physical therapy training in CMCR.

**Figure 17:**
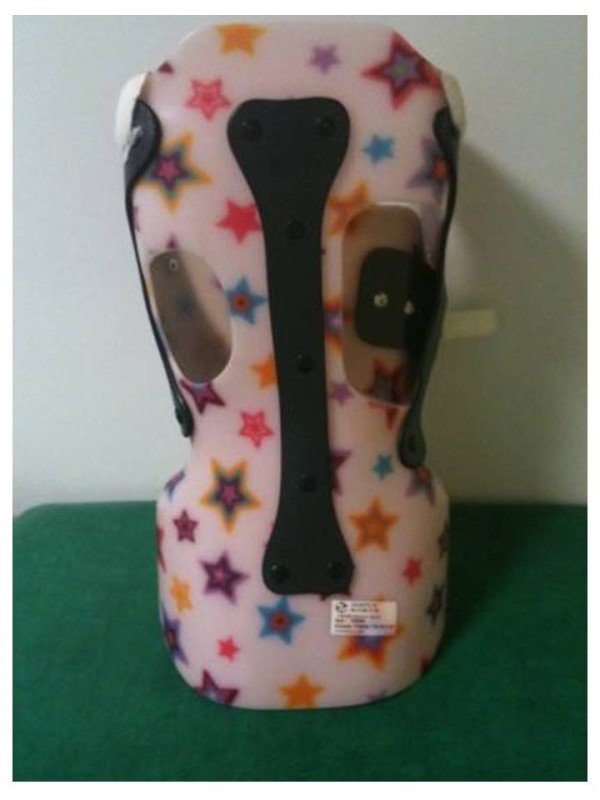
**Face and profile x-rays without brace.** Thoraco-lumbar scoliosis.

**Figure 18:**
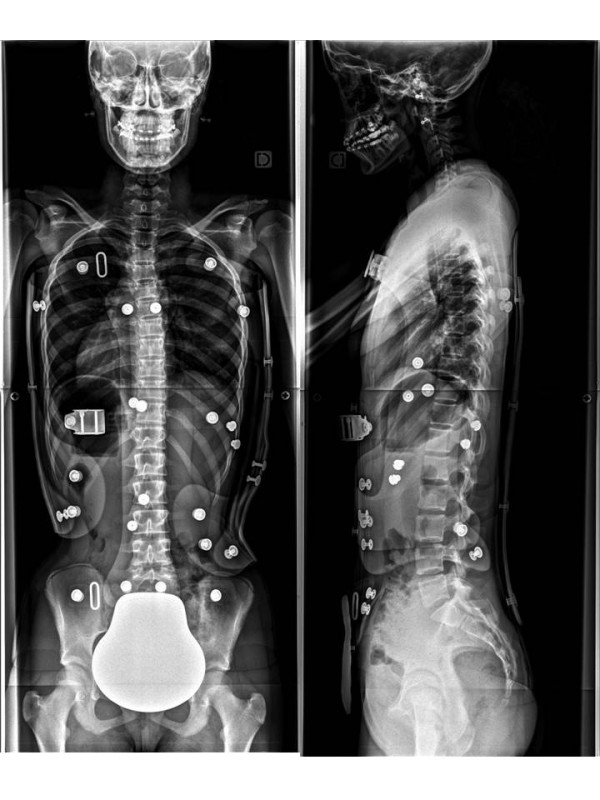
CMCR with right thoraco-lumbar pad.

**Figure 19:**
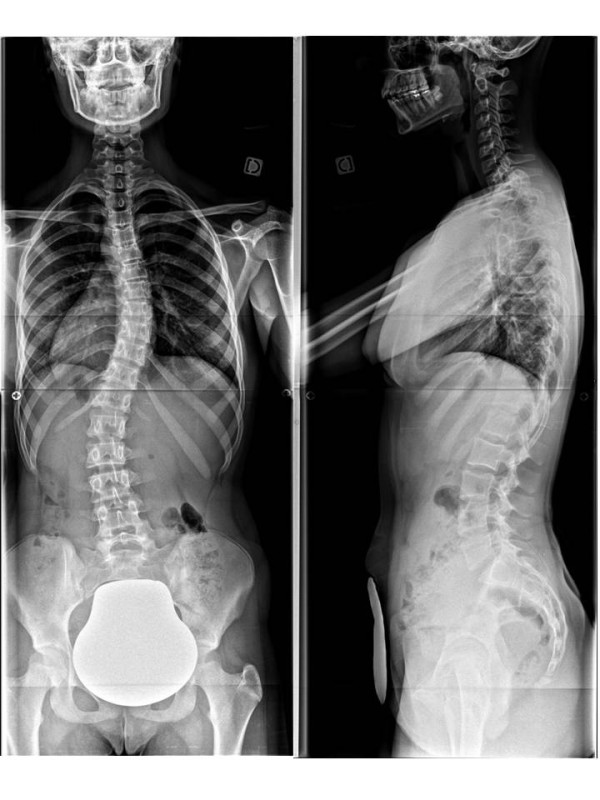
Face and profile x-raysin CMCR.

**Figure 20:**
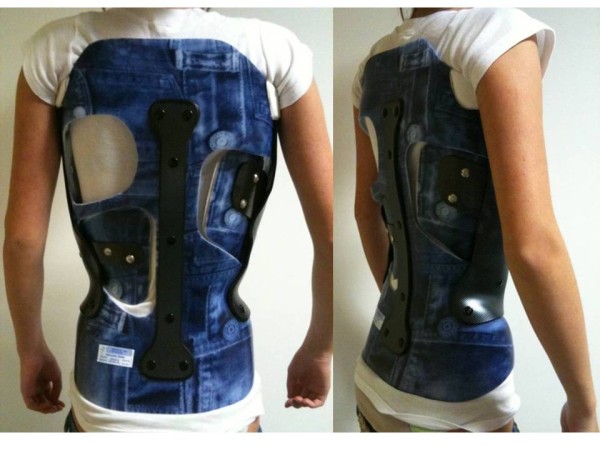
**Face and profile x-rays without brace.** Double-major scoliosis.

**Figure 21:**
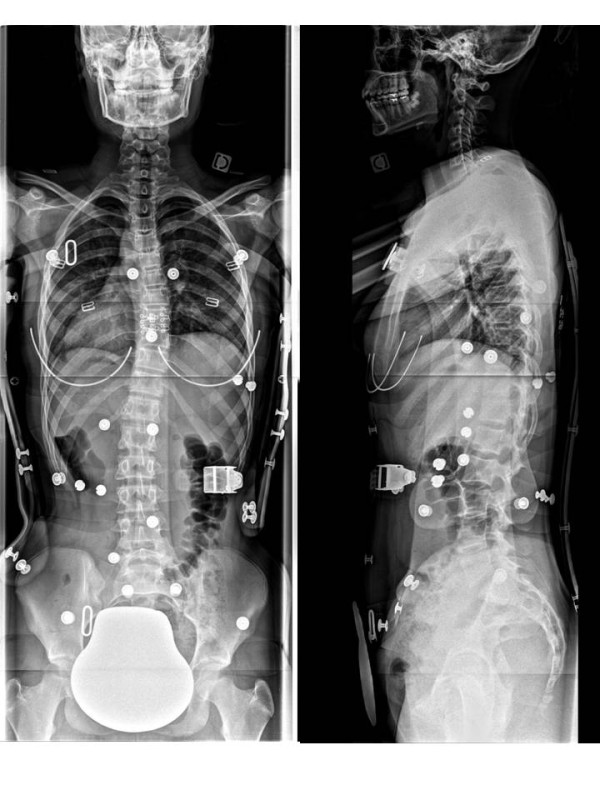
CMCR with right thoracic pad and left lumbar pad.

**Figure 22:**
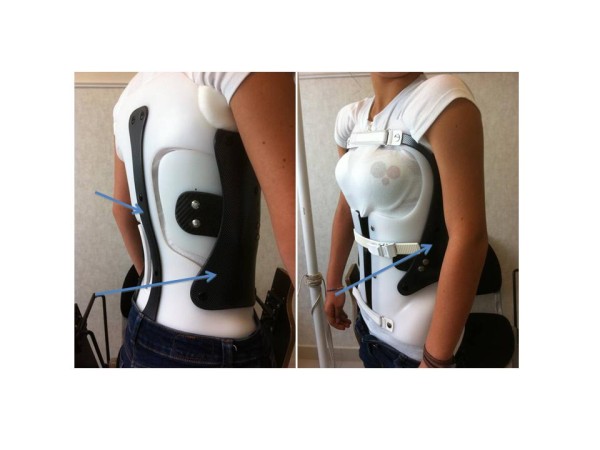
Face and profile x-raysin CMCR.

• Correction by 1 or 2 successive casts (Abbott plaster cast) made in EDF framework and worn 45 days each

• Then adaptation of a Lyon brace, worn to bone maturity.

**Table 1:** Characteristics of the population at the beginning of treatment with CMCR brace and at its definitive removal

**Variable**	** Group at the beginning of treatment**		** Group at the definitive brace removal**	
Gender	Girls	289 (90%)	Girls	86 (95%)
	Boys	32 (10%)	Boys	4 (5%)
Mean age	Girls	12 years 3 months	Girls	16 years
	Boys	11 years 1 month	Boys	15 years 5 months
Curvature type	Double-major	234 (72,9%)	Double-major	69 (76%)
	Lumbar	7 (2,2%)	Lumbar	-
	Thoracic	31 (9,7%)	Thoracic	7 (8%)
	Thoraco-lumbar	49 (15,3%)	Thoraco-lumbar	14 (16%)
Cobb angle	Double-major	21,7°	Double-major	17,6°
	Thoracic	23,6°	Thoracic	18,1°
	Thoraco-lumbar	24,1°	Thoraco-lumbar	20,2°
Skeletalmaturity	Risser 0	152 (48,4%)	Achieved	
	Risser 1	65 (20,7%)		
	Risser 2	42 (13,4%)		
	Risser 3	36 (11,5%)		
	Risser 4	19 (6,1%)		
Theoretical Vital Capacity (VC)	Girls	3,4 l.	Girls	3,5 l.
	Boys	3,5 l.	Boys	4,2 l.
Forced Vital Capacity (FVC)	Girls	2,4 l.	Girls	2,7 l.
	Boys	2,7 l.	Boys	3,6 l.

**Table 2:** Characteristics of the population at the beginning of treatment with Lyon brace

**Variable**	**Group**	**n**	**Percentage**
Gender	Girls	184	76,7%
	Boys	56	23,3%
Kind of scoliosis	Double-major	157	65,4%
	Lumbar	2	0,8%
	Thoracic	35	14,6%
	Thoraco-lumbar	40	16,7%
	Triple-curved	6	2,5%
Risser	0	58	42,3%
	1	21	15,3%
	2	21	15,3%
	3	16	11,7%
	4	13	9,5%
	5	8	5,8%

**Table 3:** Characteristics of the population at the beginning of treatment with Lyon brace and at its definitive removal

**Variable**	**Group**	**Mean at the beginning of treatment**	**Mean at the definitive brace removal**
Age	Girls	13 years 3 months	15 years 7 months
	Boys	14 years 7 months	16 years
Cobb angle	Double-major	35,5°/30,1°	31,4°/27,1°
	Thoracic	35,5°	32,6°
	Thoraco-lumbar	33,9°	31,8°
Theoretical VC	Girls	3,3 l.	3,8 l.
	Boys	4 l.	4,1 l.
FVC	Girls	2,4 l.	2,7 l.
	Boys	3,1 l.	3 l.

**Table 4:** Comparative study – Impact of the brace kind on evolution of FVC (forced vital capacity)

**Indicator**	**Groups**	**N**	**Mean**	**p-value**	<0,05 = *
FVC evolution at the brace setting up (liters)	CMCR brace	217	−0,3	0,000	*
	Lyon brace	200	−0,5		
FVC evolution at brace definitive removal compared to the beginning of treatment (liters)	CMCR brace	90	0,4	0,162	
	Lyon brace	82	0,3		

As we had to deal with earlier treatment of scoliosis, where clinical and radiological deformations are less expanded, we began to understand a bit more its evolution and were lead to develop corrective orthosis that suit to moderate scoliosis [1]. Currently available braces have demonstrated a real efficiency but still present disadvantages because of their rigidity, especially for the thoracic function that is limited by the lack of expansion while breathing in.

The purpose of this paper is to present the CMCR brace (**C**orset **M**onocoque **C**arbone respectant la **R**espiration – which means Monoshell Carbon Brace respecting Breathing).

Scoliosis that present only a lumbar component will not be considered in the brace panoply of this work.

## History

At the end of the 50’s, Pierre STAGNARA finalized his modernization and streamlining of Adolescent Idiopathic Scoliosis treatments, at Les Massues hospital. It was the time of « Lyon school » and the Lyon brace was born.

Scoliosis screening nowadays occurs earlier. Orthopaedic treatments by brace have to adapt to these lower angle deformations.

Braces should have minimal impact on these young children lives, while remaining effective on curves correction. Braces have to take into account new needs, such as comfort, lightness, aesthetic, preservation of respiratory capacity and overall respect for the child growth.

From the meeting of these new settings is born the CMCR. This monoshell brace was developed by Lecante society at the Centre des Massues [2] in Lyon in 1997, under the aegis of Dr. Bernard. Inspired by the location of the Lyon brace supports, the rigid pads are changed into mobile ones in the CMCR brace. It calls upon new materials, more efficient (x-rays transparent, dynamic…) but also more comfortable. Thus, results on scoliosis curves remain optimal without interfering with thoracic growth, and lie on a greater participation of the child, which is the key to the treatment success.

## Theoretical principles

### How the brace might work (theory)

The CMCR is a light brace reinforced by carbon blades and implemented without prior casting.

The CMCR design is based on clinical and radiological data (Cobb angles, pelvic parameters, spinal 3D analysis): they allow us to give the right direction to the force(s) implemented in the brace to correct the scoliosis torsion. To this correction force will be opposed two counter forces, to obtain a 3-points effect. It is possible to use the extension component of the CMCR, offered by the monoshell structure of the brace, by adjusting the under-axillary supports.

In a recent work [3] we have been able to show the interest of this brace, through the appropriate use of carbon material, for the rib cage development and for the respiratory capacity of these children treated with CMCR during many years, at the time where the thoracic growth is the highest.

### General description of the mechanical principles of correction

The CMCR originality lies in the appropriate use of prestressed carbon to create support forces on brace pad(s). This support keeps some mobility during movements, and especially in respiratory ones. Correction is thus permanent; the child cannot escape the support, as it is the case with rigid orthosis. The thorax mobility is preserved, sparing at best the subject breathing. Dorsal and lumbar pads can be adapted tailored to the desired correction, and considering the 3 plans. The brace will be individualized regarding curve type, subject age and weight, trying to get the best compromise for the patient, throughout treatment.

#### The classification used for prescription

The treated group is made of 90% girls and 10% boys (sex ratio) which corresponds to the usual prevalence data in the literature about scoliosis (Figure 1).

The CMCR is implemented at 12 years on average; a little younger in boys (11 years) than in girls (12 years).

It is generally worn for progressive scoliosis with a Cobb angle from 20 to 30°, but can be implemented in some other cases, taking into account the 3D analysis [4], patient age and sitting size, and curve(s) reducibility.

### Variations of the brace according to the curve pattern: description for the 3 kinds of curves treated with CMCR

The CMCR is prescribed for combined scoliosis (or double major) in more than 73% of cases. Thoraco-lumbar scoliosis represents 15%, and finally 10% of thoracic ones.

This sharing out is not due to any technical specificity of the brace, but corresponds to the percentages of scoliosis various forms in our everyday practice. We need to state that no lumbar scoliosis is treated with CMCR, because we use another orthosis (3-points brace) for this kind of scoliosis.

Combined scoliosis: the brace is equipped with two mobile pads.

• One pad on the dorsal deformity: after 3D analysis, the orientation of the support force corrects the torsion, with a maximal orientation ¾ antero-posterior when the torsion is maximal. Prestressed carbon of the mobile pad allows by its elasticity the inspiratory movements and promotes the expiratory ones.

• One pad on the lumbar deformity: the support force of this pad is diametrically opposed to the thoracic one to facilitate spinal detorsion. If needed, this lumbar pad will render some lordosis by pressing on vertebrae transverse apophysis (Figure 2).

Thoracic scoliosis: the brace is equipped with only one pad on the dorsal deformity, associated with two counter-supports; one superior under-axillary counter-support on the opposite side to the thoracic curve and another inferior counter-support next to the lumbar column, still opposite to the thoracic curve.

The deformation 3D correction is made by adjusting precisely the location and the pressure of the pad, as explained before.

The pad will be all the more lateral that the torsion is important; in this case, it is no more directly on the hump noticed by clinical examination. The more anterior is the pad, the more necessary it is to put another mobile pad (and not only a simple rigid counter-support) to avoid the lumbar kyphosis.

Adaptation difficulties: the CMCR is sometimes difficult to adapt in high thoracic scoliosis, with a few place for the superior counter-support.

Thoraco-lumbar scoliosis: the brace is equipped with an only pad on the thoraco-lumbar deformity, associated with two rigid counter-supports. Be careful not to disturb the sagittal plane (as can be seen with the hyper corrective brace) as the main pad must allow a dual objective: regain some dorsal kyphosis and some lumbar lordosis. It is only by the 3D analysis that the pad correct orientation can be found and then proved when the brace adaptation is completed.

## Brace description

The CMCR is a monoshell brace with an innovative system of mobiles pads. It preserves vital capacity by mobile pressures in thoracic and in lumbar, thanks to use of prestressed carbon. It retains pads from the Lyon brace, but gives more opportunities to orient forces [5].

The basic structure is in polyethylene. There is an anterior opening to allow an easier putting on (Figure 3).

Three strengthening pieces of Kydex (KYDEX sheet is an acrylic-polyvinyl chloride composite produced by KYDEX, LLC. Engineered for thermoforming fabrication, KYDEX sheet, combines the advantageous properties of both the acrylic and the polyvinyl chloride components. From acrylic, it obtains superior rigidity and formability; from PVC, outstanding toughness, chemical resistance and good interior finish ratings) plasticstiffen this shell: one medial posterior that stiffens the sagittal plane and two lateral ones that set in addition the carbon blade.

### The Main shell

Lumbar pad: (if needed) (Figures 4 &5)

It is independent of the main shell and provides lateral translation. The pressure of the pad is lateral and paravertebral (to correct lumbar rotation and to maintain lumbar lordosis).

The carbon blade is the link between the brace and the pad (slipped under the lateral reinforcement and fixed on it).

This attachment device allows variations of strength and/or location of the pad.

#### *Thoracic or thoraco-lumbar pad*

This shell is also independent and is built like the lumbar pad (Figure 6). Its position has to be lateral and a bit posterior, to allow the thoracic spine to stay in kyphosis and avoid the flat back.

The carbon blade is the link between the brace and the pad; its suppleness allows rib cage movements for a good breathing. (It is slanted to follow the ribs inclination).

This attachment device allows variations of strength and/or location of the pad.

#### *Carbon blades*

These blades are made of carbon fibre braids (mainly unidirectional).Their thickness, determined by the number of layers, gives the searched flexibility according to every type of patients.

The carbon blade is prestressed and *keeps pressure during exhalation*.

#### *Fastening systems*

The main system of opening and closing is a flexible rack at chondro-costal awnings level to maintain a constant clamping. Velcros at manubrial and pelvis levels complete it.

#### *Finishing touches*

Foam is added on pressure area and sub axillary. A leather flap is placed on the opening of the brace, between the two polyethylene parts.

## Pratical issues

### How to prescribe the brace: principles of correction written in prescriptions by MDs

The CMCR is prescribed after a clinical and radiological examination of the patient. The prescription takes into account patient age, scoliosis progression and deformity reducibility. Here is the standard prescription form for a combined scoliosis:

• CMCR brace for combined scoliosis right thoracic/ left lumbar with:

∘ Right dorsal pad T5-T12. *Specify the orientation that should be given to the pad*: ¾ *posterior or lateral*.

∘ Left lumbar pad T12-L4. *Specify the orientation that should be given to the pad*: *postero*-*anterior or lateral*.

∘ Rigid left sub-axillary counter-support.

∘ Control of the chondro-costal awnings, with kyphosis effect if necessary.

### How to build the brace: **principles** of construction by CPOs, with some photos; (see also the discussion regarding brace pressure above

#### Optical acquisition

Lecante society uses since 1997 ORTEN® system for the manufacture of CMCR (Figure 7). This is an optical 3D image acquisition method for the trunk, and a *Conception et Fabrication Assistée par Ordinateur* (CFAO) software (Cad cam system) for the rectified positive [6-8].

The sensor consists in a portico of 2 meters side square in the center of which the patient is placed. 4 CDD camera recorders are symmetrically distributed in 4 columns, as well as 8 structured light projectors that project alternatively black and white fringes on the patient. The deformation of these fringes allows the calculation of the bust outer surface.

The acquisition is completed within less than 2 seconds (which allows an optimal posture).

Its accuracy is of the order of one millimeter.

#### Positioning the patient

The subject is covered with a white tubular slinky jersey (Figure 8).

It is then placed at the center of the sensor, elbows apart and hands forward at shoulder height, on two vertical stands.

This position is checked on the computer screen and can be modified prior to the optical print acquisition (Figures 9 &10).

#### Rectifying the positive

The optical acquired data are then rectified on computer by the orthotist, in the same way as for a plaster positive (Figure 11).

At this stage we realize the brace final form with expansion areas and pressure areas on deformities, as well as the creation of the waist that will be the brace base).

Data is then sent to a milling machine that carves the positive in polyurethane foam (Figure 12).

#### Thermoforming the trial brace

Thermoforming of polyethylene (PE) is made at 160° by manual draping on the foam positive. The welding of PE plate is made on the front part of the positive (since the opening of the brace is anterior). The PE cools all night long, and the brace is removed from the positive the next morning.

#### Fitting

This molded trial brace is then put on the patient, as if it were the final brace (it should not be too big not to hurt the patient, and should allow to keep a precise vision of the definitive brace). It is maintained closed with tape.

Fitting helps to:

• Control volumes adaptation

• Define the cutting on different sides of the brace : anterior (chest), superior (clavicles), and inferior (in the groin)

• Determine the location of the pads (with help of radiological and clinical examination)

• Determine the place of carbon blades as well as the power that will be applied on pads (depending on the thickness and the width of the blade)

• Determine where the Kydex® reinforcements should be placed

• Precise the position of the expansion areas

• Notice potential painful points (Figure 13)

#### Finishing touches

Brace and pads are cut (Figure 14).

We realize then 3 Kydex® reinforcements (a posterior one and 2 side ones), which are coupled to the brace by rivets.

Carbon blades and pads are then assembled (Figure 15).

#### Brace delivery

Still checking radiography, the orthotist puts the brace on the child who wears a cotton seamless shirt.

He makes sure that the brace is well adapted, with all parts on the right height. The fitting depends on the scoliosis importance and reducibility. The child should not be too embarrassed by its brace, and especially the sitting position should be paid attention to.

### How to check the brace**:** principles of checking by MDs and CPOs

Before starting treatment, the patient and its parents receive oral and written explanations on the kind of brace and on the different steps of its manufacture and adaptation.

To be enlightened before making the choice of treatment, the patient receives as well oral and written information about inconvenience that might occur during treatment (as for example cutaneous irritation, sleeping troubles, constriction feeling…) and means to prevent it.

CMCR-wearing patients and their parents benefit throughout treatment from a therapeutic education to be able to manage treatment by themselves.

The child is seen every morning during one week to adapt brace, adjust carbon pressure, and improve comfort and quality of life in orthosis.

A front/profile radiography is done at the late brace adaptation to control its effectiveness (especially to confirm that all supports are well-oriented and at the right height). This x-ray is compared to x-ray without brace as well as face x-ray with partial suspension [9,10].

In the making and at the brace delivery, simple explanation is given to get the child and its parents understand how the brace works.

• Fitting tips: indelible marks are put on brace straps to help along.

• Tips to put on and off the brace without help, which is part of the therapeutic education: either in a standing position or in a lying one.

• Cleaning tips: brace is worn on a seamless T-Shirt that should be changed every day. Brace has to be washed with soap once a week.

• In case of technical problem: manufacturer and referent physiotherapist at Les Massues can be joined by phone. Address and telephone number of the fabricant workshops are given, depending on where the patient lives, to correct technical problems if necessary.

### Protocols: description of the protocols generally used according to each clinical situations; (see criteria for bracing)

Three months after brace adaptation, the patient is first seen by the referent physiotherapist at Les Massues, who assesses how the brace is physically and psychologically tolerated, how respiratory capacity, height and weight evolve. The patient benefits then from a clinical examination by the senior physician doctor (codified clinical examination without brace) and meets afterward the orthotist. All this controls are carried out in a multidisciplinary united consultation at day hospital.

After 6 months of brace wearing, these controls are made again besides a face/profile naked standing radiography which is compared to the initial one.

Successive checking is made every 6 months with naked face radiography.

The brace renewal is proposed when growth no longer allows a correct adaptation of the orthosis (cutaneous or bone conflicts impossible to resolve). We cannot adapt the distance between pelvic girdle and sub-axillary supports which is an advantage of Lyon brace.

A synthetic form of clinical and radiological patient data (“spine sheet”) is regularly supplemented to have an insight into scoliosis evolution at a glance.

### Everyday usage: the number of hours per day that the patient will wear the brace

During pubertal growth period, brace is worn on average 20–21 hours out of 24, with permission to take it off for sport practice at school and outside school.

When the peak of pubertal growth has gone away (at least 18 months after the first menstrual period), and if the improvement is stabilized concerning weight and sitting height, a gradual removal is started…until a night wearing the last 6 months of treatment. At that time, sitting height, standing height and weight are stable since about one year.

Before the pubertal growth and if scoliosis is moderate and supple (less than 25°), brace will not be worn at school but will be worn around 18 hours from school end until the next morning.

### Exercises: specific exercises while in the brace (if any), with photos of how to perform them

• Getting aware of deformity (Figure 16)

• Breathing work: getting aware of abdominal and diaphragmatic breathing, fight against restrictive syndrome

• Trying to get more spinal mobility: asymmetric exercises to open concavity(ies) and ilio-lumbar angle (stretching), fight against the increase of sagittal deformities (erasing or accentuation of curves), active axial self-extension (symmetrical stretching and muscles developing in long position)

• Harmonization of anterior and posterior muscles: spinal muscles developing in long position, abdominal strengthening (chondro-costal awnings, pelvic tilt, …)

• Upkeep of sub-pelvic extensibility and musculature: upkeep of sub-pelvic muscles extensibility (growth), strengthening exercises to compensate for the lack of mobility caused by the orthosis

• First sight of relaxation

## Results

This study is retrospective among 321 patients treated in our department, which explains that the group is made of 321 patients at the beginning of the study, but of only 90 patients at the end of the study, because only 90 patients at that time had completed the treatment [3,11,12] (Table 1).

At the definitive brace removal, all kinds of scoliosis are stabilized by the orthosis and VC has increased. One may infer that the patient presents at the end of treatment a low residual deformity, which is unlikely to keep on getting worse in adulthood.

## Case reports

### Case n°1

Alice 12 years old

Thoraco-lumbar curve T6L2 30° right

T6L2 6° right in brace (Figures 17,18 &19)

### Case n°2

Camille 13 years old

Double major T4T11 28° right T11L4 29° left

In brace: T4T11 21° right T11L4 13° left (Figures 20,21 & 22)

## Discussion

Later in this work we compare the group treated with CMCR to a group of patients treated with Lyon brace [13].

The characteristics of the scoliotic group treated with Lyon brace are presented in the table below about 240 patients. Kinds of scoliosis are similar to the CMCR group (Table 2).

Lyon brace is used later that CMCR to treat scoliosis: 2 years later on average in girls and 3 years later on average in boys.

Cobb angle at the beginning of treatment is 10° more on average in Lyon brace compared to CMCR; VC without brace is reduced by 28% from the theoretical VC (Table 3).

At the final removal of Lyon brace, curves are stabilized. However, residual Cobb angle is over 30°, which means that scoliosis will have to be regularly controlled, even in adulthood, to make sure that scoliosis evolution will not continue (Table 4).

As shown by the table below, VC decreases less at the CMCR setting up (−0.3) than at the Lyon brace setting up (−0.5) and *this difference is significant*.

At definitive brace removal, VC has more increased in CMCR (0.4 liter on average) than in Lyon brace (0.3 liter on average) but *this difference is not significant*.

The Lyon brace setting up must be preceded by a reduction by Abbott plaster to reduce curve importance. Scoliosis treated by CMCR are generally less pronounced; however it can happen that in our daily practice we begin treatment with a scoliosis reduction cast, followed by a CMCR if the obtained correction is sufficient to be maintained by CMCR carbon supports.

The Lyon treatment can only be started around puberty whereas CMCR can be used whatever the age.

This leads us to propose several kinds of brace (CMCR, Milwaukee brace, Lyon brace, hyper-correcting brace) for our scoliotic patients to make a choice depending on patient age, importance of scoliosis…

It is not possible to treat all kinds of scoliosis with an only brace.

## Conclusion

CMCR brace is innovative for several reasons:

• The use of prestressed carbon corrects deformity without blocking chest movements and thus preserves respiratory capacity in brace.

• The scoliosis correction is progressive through the use of mobile supports which can always be adapted to the stiffness areas appearing throughout growth.

• Radiological analysis with 3D reconstruction is used before brace design and at the late adaptation to get an optimal scoliosis correction by using minimal support forces.

3D analysis of scoliosis is essential to allow a gradual return to a harmonious cervical, dorsal and lumbar profile.

It has been demonstrated that CMCR brace allows a better quality of life than Lyon brace [14,15]. Orthopaedic treatment is still a heavy treatment for adolescents in growth period; this orthosis is designed to preserve partly spine and chest mobility.

We hope so to have part in improving life conditions of these teenagers, compared to those treated with rigid braces.

## Competing interest

No conflict of interest.

## Consent

The patients who participated in this work and, in particular, the patients shown in the iconography gave their written agreement as well as their parents.

## Ethical approval

This study was approved by the Ethics Committee of the Massues’Center- Croix-Rouge française.
